# KDM4B-mediated epigenetic silencing of miRNA-615-5p augments RAB24 to facilitate malignancy of hepatoma cells

**DOI:** 10.18632/oncotarget.10832

**Published:** 2016-07-25

**Authors:** Zheng Chen, Xiangling Wang, Ruiyan Liu, Lin Chen, Jianying Yi, Bing Qi, Zeyu Shuang, Min Liu, Xin Li, Shengping Li, Hua Tang

**Affiliations:** ^1^ Tianjin Life Science Research Center and School of Basic Medical Sciences, Tianjin Medical University, Tianjin, China; ^2^ Department of Laboratory Medicine, the First Teaching Hospital, Tianjin University of Traditional Chinese Medicine, Tianjin, China; ^3^ State Key Laboratory of Oncology in Southern China, Department of Hepatobiliary Oncology, Cancer Center, Sun Yat-sen University, Guangzhou, China

**Keywords:** DNA methylation, miRNAs, metastasis, HCC, gene regulation

## Abstract

Emerging evidence indicates that dysregulation of microRNAs (miRNAs) contributes to hepatocellular carcinoma (HCC) tumorigenesis and development. Here, we found that miR-615-5p was obviously downregulated in HCC. Furthermore, the deficiency of demethylase KDM4B stimulated the CpG methylation of miR-615-5p promoter and then decreased the miR-615-5p expression. The Ras-related protein RAB24 was found to be downregulated by miR-615-5p. The low level of miR-615-5p increased the expression of RAB24 and facilitated HCC growth and metastasis in vitro and in vivo. Moreover, miR-615-5p suppresses HCC cell growth by influencing cell cycle progression and apoptosis. Downregulation of miR-615-5p and upregulation of RAB24 promotes the epithelial-mesenchymal transition (EMT), adhesion and vasculogenic mimicry (VM) of HCC cells, all of which contribute to cell motility and metastasis. Thus, miR-615-5p, who is downregulated by KDM4B-mediated hypermethylation in its promoter, functions as a tumor suppressor by inhibiting RAB24 expression in HCC. In conclusion, our findings characterize miR-615-5p as an important epigenetically silenced miRNA involved in the Rab-Ras pathway in hepatocellular carcinoma and expand our understanding of the molecular mechanism underlying hepatocarcinogenesis and metastasis.

## INTRODUCTION

HCC is one of the most prevalent and lethal cancers worldwide [1]. Despite the serious consequence of this disease, we have only an elementary understanding of the molecular mechanisms underlying its pathogenesis [2]. In recent years, an increasing number of reports have described a new class of small regulatory RNAs, termed microRNAs (miRNAs), that are implicated in the pathogenesis of several cancers, including HCC. However, little is known about the causes of the widely differential expression of miRNAs between cancer and normal cells. In cancer, CpG islands of promoter are commonly hypermethylated, and the methylation is often associated with repression of the target gene [3]. In fact, approximately 20% of all miRNAs are embedded within CpG islands [4]. DNA methylation plays a key role in the silencing of numerous miRNA encoding genes, suggesting the existence of tumor-suppressive miRNAs that are epigenetically downregulated in HCC. In this work, HepG2 cells were treated with 5-Aza-CdR, and 48 out of the 640 human miRNAs examined were upregulated. In particular, we focused on miR-615-5p because it was the most strongly induced by 5-Aza-CdR treatment and the mechanism by which it promotes the hepatocarcinogenesis is relatively unknown. DNA methyltransferases enzymes (DNMTs) are directly responsible for the hypermethylation of CpG islands in promoters. However, DNA methylation is closely associated with histone modification due to the cross-talk mediated by the methyl-CpG-binding domain proteins (MBDs) [5]. Thus, methylation-associated molecules, including DNMTs, MBDs, HDACs, HMTs and HDMs, may play important roles in the silencing of the miR-615-5p gene. Although someone had investigated the expression of miR-615-5p in HCC [6], they did not discuss the relationship between DNA methylation and miR-615-5p expression and the underlying mechanism through which miR-615-5p regulate HCC cell growth and metastasis.

Properties related to cancer development and aggressiveness include the ability to maintain proliferative signaling, activate metastasis and induce angiogenesis [7]. Increasing evidence suggests that miRNAs participate in nearly every step of the pathogenesis of cancer. Therefore, it is possible that miR-615-5p affects the malignant phenotypes of HCC. It is well known that epithelial-mesenchymal transition (EMT) can disrupt intercellular contacts, enhance cell motility and facilitate the release of cancer cells from the primary tumor. In addition, the metastatic mechanism also includes the interaction between tumor cells and microenvironment at secondary sites, such as cell-matrix adhesion. However, the relationship between miR-615-5p and EMT, cell adhesion is not clear.

RAB24 is a member of the Rab subfamily of Ras-related proteins that regulate intracellular protein trafficking [8]. It has been reported that three members of the Rho family of small GTPases-RhoA, Rac1 and cell division cycle 42-are crucial in regulating the signaling pathways involved in cytoskeletal remodeling, cell morphology, motility and adhesion [9, 10]. However, the role of RAB24 in regulating HCC cell motility and adhesion remains to be elucidated.

Here, we performed both microarray and qRT-PCR to investigate the epigenetic silencing of miR-615-5p in clinical HCC samples and the cell lines. We found that downregulation of KDM4B mediated hypermethylation of the miR-615-5p promoter. miR-615-5p suppressed the HCC cell growth, migration, invasion and adhesion. Moreover, RAB24 was identified as a functional target of miR-615-5p. Collectively, the present work provides the first evidence of the coordination of methylation-modulated miR-615-5p and RAB24 in the regulation of the cell cycle, apoptosis, EMT and the β1-integrin pathway during hepatocarcinogensis.

## RESULTS

### miR-615-5p is hypermethylated and silenced in HCC

**Figure 1:**
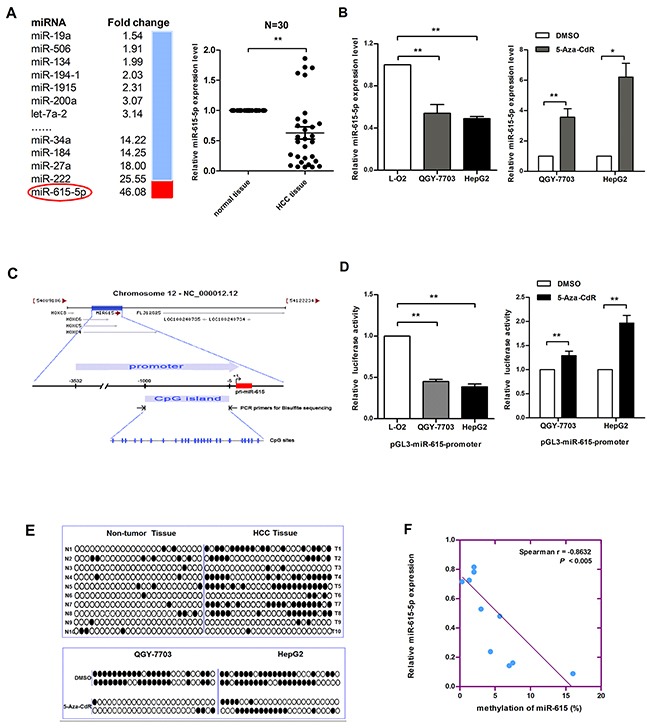
Promoter DNA hypermethylation mediates the downregulation of miR-615-5p expression in HCC **A**. The fold changes in the levels of miRNA in HepG2 cells treated with 5-aza-CdR was determined by microarray analysis (left). The expression level of miR-615-5p in 30 pairs of HCC tissues was measured by qRT-PCR, U6 snRNA was used for normalization (right). Error bar indicate mean±SEM, *, p<0.05. **B**. The mRNA level of miR-615-5p in HCC cell lines (left) and after treatment with 5-Aza-CdR (right) was measured by qRT-PCR. **C**. The diagram shows the promoter region of the miR-615 gene and the CpG island located within this region. The blue vertical bar represents the CpG sites. **D**. Luciferase reporter system was used to detect the promoter activity of miR-615-5p in HCC cell lines (left) and after 5-Aza-CdR treatment (right). **E**. The black circle indicates methylated CpG loci and the white circle indicates unmethylated CpG loci. **F**. Scatter plots showing miR-615-5p expression compared with methylation. Error bars in (B) and (D) indicate the mean±SD of three independent experiments. *, p<0.05, **, p<0.01.

To screen for miRNAs that may be regulated by DNA methylation in HCC, we treated HepG2 cells with 5-Aza-CdR, which is frequently used to induce demethylation. Next, we examined the expression level of 640 miRNAs by microarray analysis. The results showed that 48 miRNAs (Supplementary Table S1) were upregulated and 27 miRNAs (Supplementary Table S2) were downregulated. We chose miR-615-5p for further study because its expression was the most strongly upregulated (Figure 1A). To confirm the downregulation of miR-615-5p in vivo, we detected the expression level of miR-615-5p in 30 pairs of clinical HCC samples by qRT-PCR. As expected, the level of miR-615-5p in HCC tissues was significantly lower than that in adjacent normal tissues (Figure 1A). In HCC cell lines QGY-7703 and HepG2, the expression of miR-615-5p was lower than that in the normal human liver immortalized cell line L-O2 (Figure 1B). Treatment with 5-Aza-CdR restored the expression of miR-615-5p (Figure 1B), which was consistent with the results obtained with the microarray (Figure 1A).

To verify the effect of DNA methylation on miR-615-5p expression, we cloned a fragment with promoter activity (−3 532 to −5 upstream of miR-615-5p) (Supplementary Figure S1) into the pGL3-Basic vector, and we found a CpG island harboring 25 CpG dinucleotides (−1 000 to −5) in this promoter region (Figure 1C). The luciferase reporter assay revealed that the promoter activity of miR-615-5p in HCC cell lines was lower than that in L-O2 cells, and 5-Aza-CdR treatment restored its activity (Figure 1D). Next, genomic bisulfite sequencing was performed to determine the methylation status of the miR-615-5p promoter in 10 pairs of HCC tissues (T1-T10) and cell lines. The results revealed that the methylation level was higher in HCC tissues than in normal tissues (Figure 1E). Meanwhile, miR-615-5p was highly methylated in QGY-7703 and HepG2 cells, and the methylation level decreased after 5-Aza-CdR treatment (Figure 1E). The relationship between methylation and expression can be demonstrated by analyzing the correlation between the genomic DNA and RNA isolated from the same patient. Spearman's rank correlation analysis revealed an inverse correlation between methylation and the expression of miR-615-5p (Figure 1F). These results suggest that miR-615-5p is epigenetically downregulated in HCC.

### Downregulation of KDM4B mediated DNA hypermethylation, thereby inducing miR-615-5p silencing

**Figure 2:**
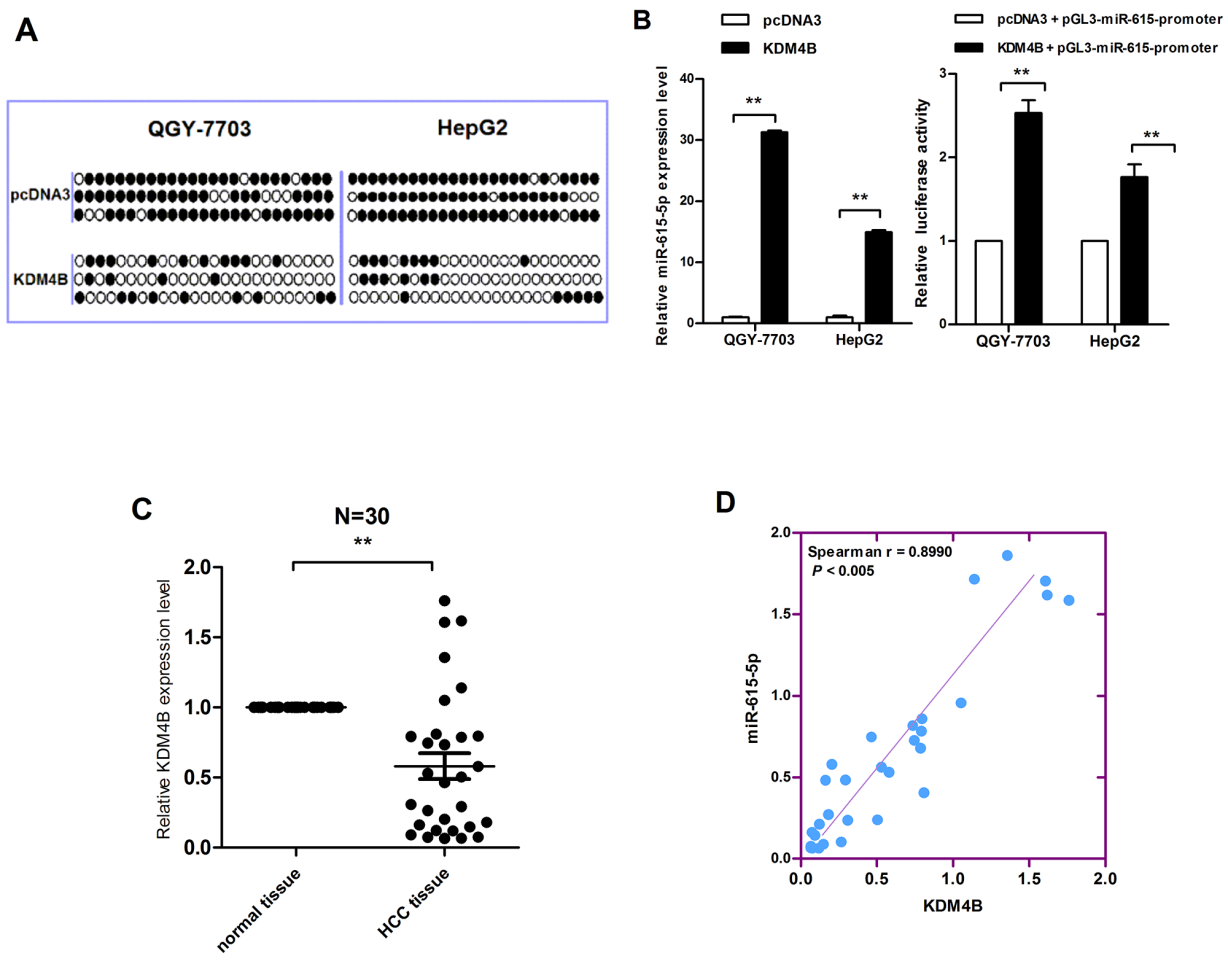
The correlation between KDM4B downregulation and miR-615-5p silencing **A**. Genomic bisulfite sequencing revealed the methylation status of HCC cells transfected with KDM4B overexpression vector and control vector. **B**. The mRNA level and the promoter activity of miR-615-5p after overexpression of KDM4B in HCC cells was determined by qRT-PCR (left) and luciferase reporter assay (right). Error bars indicate the mean±SD of three independent experiments. **, p<0.01. **C**. The mRNA level of KDM4B in 30 pairs of HCC tissues was measured by qRT-PCR, β- Actin was used for normalization. Error bar indicate the mean±SEM, *, p<0.05. **D**. The correlation between miR-615-5p and KDM4B expression.

Because the miR-615-5p promoter is hypermethylated in HCC, we hypothesized that the deregulation of a specific methylase or demethylase induces this process. To identify the putative methylase/demethylase responsible for miR-615-5p methylation, several expression vectors, including vectors harboring DNMT1, DNMT3a, KDM4A, KDM4B, KDM4C, KDM5A and KDM6A, were transfected into QGY-7703 cells respectively (Supplementary Figure S2). A detailed analysis by bisulfite sequencing indicated that only the vector harboring KDM4B significantly reduced the number of methylated CpG sites (Figure 2A). Therefore, we hypothesise that KDM4B is involved in the DNA methylation-mediated silencing of miR-615-5p. Indeed, the mRNA level and promoter activity of miR-615-5p was recovered when KDM4B was ectopically expressed in HCC cells (Figure 2B). We also examined the expression level of KDM4B in 30 pairs of clinical HCC tissues. The results revealed that, KDM4B was generally expressed at a lower level (Figure 2C). Spearman's rank correlation analysis revealed a positive correlation between KDM4B and miR-615-5p expression (Figure 2D). The above results suggested that downregulation of KDM4B could directly induce the hypermethylation of miR-615-5p.

### miR-615-5p suppresses HCC cell growth by delaying cell cycle progression and facilitating apoptosis

**Figure 3:**
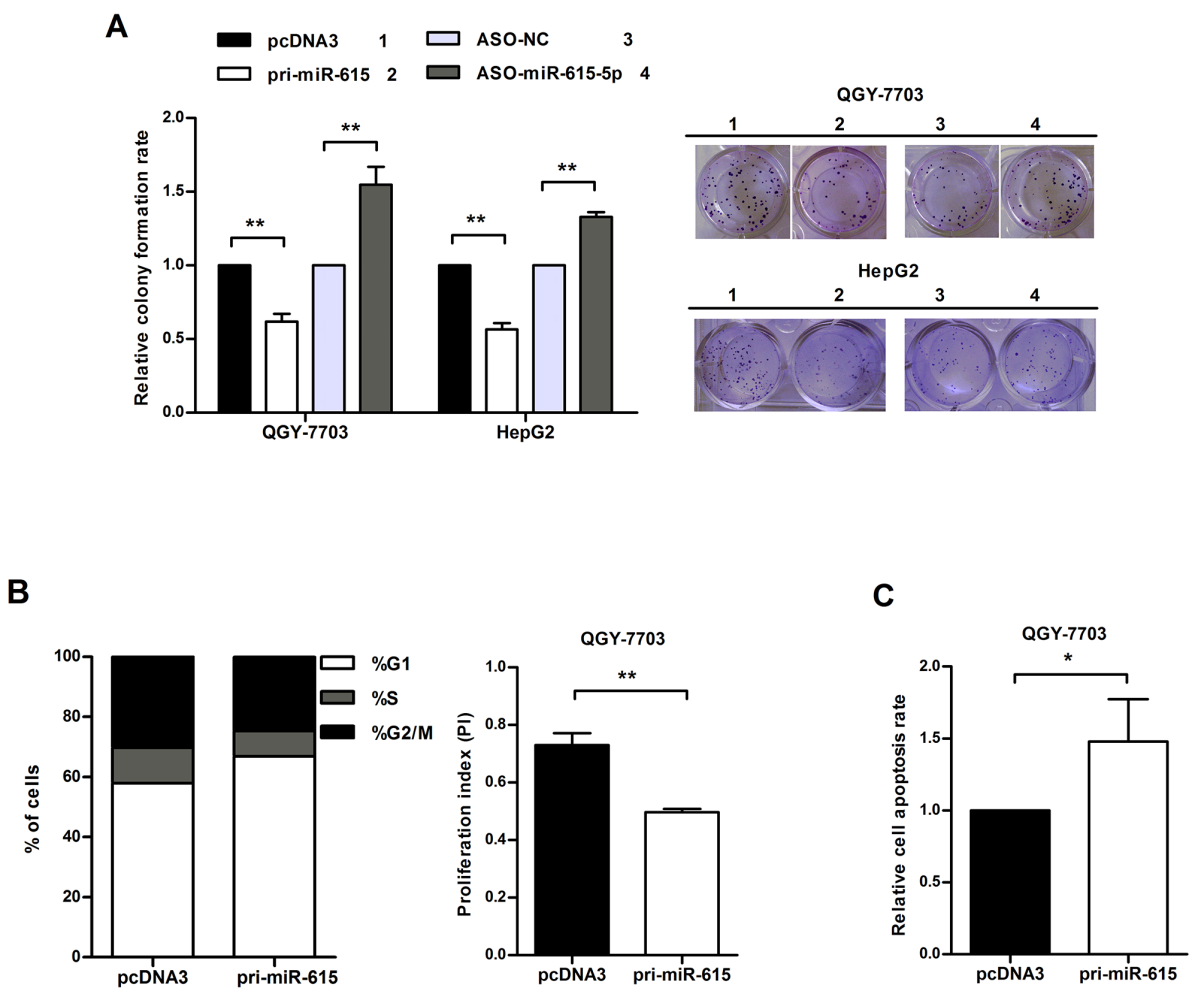
miR-615-5p suppresses HCC cell growth by blocking cell cycle progression and activating apoptosis **A**. Colony formation rate = (number of colonies/number of seeded cells) × 100%. **B**. The proliferation index (PI) was calculated with the equation: PI = (S+ G2/M) / G1 (S, G2/M and G1 indicate the percentages of cells in S, G2/M and G1phase, respectively). **C**. Apoptotic cells in three random fields were counted, and the apoptosis rate was calculated. Error bars in (A), (B) and (C) indicate the mean±SD of three independent experiments. *, p<0.05, **, p<0.01.

To determine the effects of miR-615-5p on the malignant behaviors of HCC cells, a miR-615-5p overexpression vector, pcDNA3/pri-miR-615 (pri-miR-615) and a commercially synthesized 2’-O-methoxyl-modified antisense oligomer (ASO-miR-615-5p) were used to alter the expression level of miR-615-5p in HCC cells. After the efficiency of these vectors was confirmed by qRT-PCR (Supplementary Figure S3), we investigated the effect of miR-615-5p on cell growth using a colony formation assay. The result showed that the colony formation rate of QGY-7703 cells transfected with pri-miR-615 was decreased by approximately 39% compared with the control group, whereas transfection with ASO-miR-615-5p increased the colony formation rate by approximately 1.54-fold (Figure 3A). Similar results were obtained in HepG2 cells (Figure 3A).

To further evaluate the suppression in cell growth caused by miR-615-5p, we performed flow cytometry to detect the impact of miR-615-5p on cell cycle. Overexpression of miR-615-5p led to an increase in the proportion of cells in G1 phase (from 58% to 67%) and a decrease in the proportion of cells in S phase (from 12 % to 8%) (Figure 3B). The proliferation index (PI) of the miR-615-5p overexpression group was lower than the control group (Figure 3B). Meanwhile, overexpression of miR-615-5p led to an increase in the apoptosis rate (Figure 3C). Therefore, we conclude that miR-615-5p suppresses HCC cell growth by slowing down cell cycle progression and accelerating apoptosis.

### miR-615-5p inhibits cell motility, cell adhesion and vasculogenic mimicry formation in HCC

**Figure 4:**
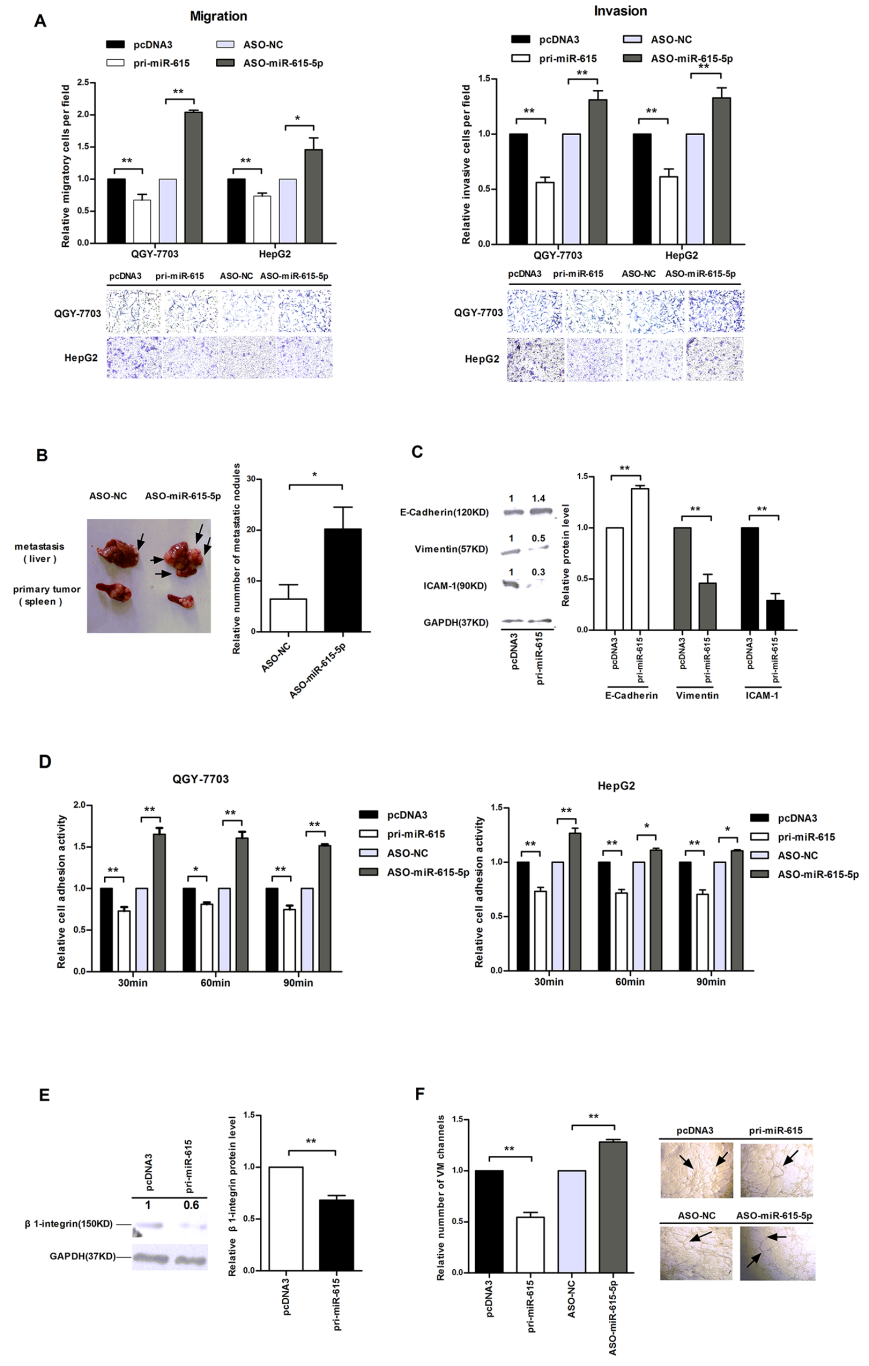
The influence of miR-615-5p on HCC cell motility, adhesion and vasculogenic mimicry **A**. Transwell migration (left) and invasion assay (right) of HCC cells. Cells in three random fields of view at 100x magnification were counted and expressed as the average number of cells per field. A representative image is shown below. **B**. In the in vivo xenograft mouse model, the tumor nodules in primary sites (spleen) and metastatic sites (liver) 6 weeks after transplantation are shown. Black arrows indicate the location of metastatic nodules. The numbers of intrahepatic metastatic nodules in each mouse were counted, and the quantification is shown. **C**. The protein levels of E-cadherin, vimentin and ICAM-1 in QGY-7703 cells after transfection with pri-miR-615. **D**. The absorbance was measured at a wavelength of 570 nm at 30, 60 and 90 min after seeding in the cell adhesion assay. **E**. The protein level of β1-integrin when miR-615-5p was overexpressed. **F**. Vasculogenic mimicry assay in QGY-7703 cells. Error bars in (A), (C), (D), (E) and (F) indicate the mean±SD of three independent experiments. *, p<0.05, **, p<0.01.

To explore whether miR-615-5p can also affect HCC cell motility, transwell migration and invasion assays were performed. The results revealed that the migration ability of QGY-7703 and HepG2 cells transfected with pri-miR-615 was decreased by approximately 27%-33%, whereas cells transfected with ASO-miR-615-5p presented an approximately 1.46-2.0 fold increase compared to the corresponding control (Figure 4A). The transwell invasion assay showed a similar result (Figure 4A). These data indicate that miR-615-5p suppresses HCC motility in vitro.

To further confirm the effect of miR-615-5p on HCC metastasis in vivo, we implanted QGY-7703 cells transfected with ASO-miR-615-5p or ASO-NC into the upper pole of the spleen in 11 pairs of nude mice. Intrahepatic metastasis nodules were detected in 8 mice in both groups. The number of metastatic nodules in liver was dramatically increased when the miR-615-5p level was reduced (Figure 4B). Hematoxylin-Eosin (HE)-staining revealed the loci of the metastatic tumors derived from cells transfected with ASO-miR-615-5p (Supplementary Figure S4). To verify that the increase in the number of metastatic nodules was caused by miR-615-5p reduction, we detected the level of miR-615-5p in the metastatic tumors. The miR-615-5p level in ASO-treated group was lower than that in ASO control group (Supplementary Figure S5). These data revealed that downregulation of miR-615-5p promoted the HCC metastasis in vivo.

To explore whether miR-615-5p modulates EMT to influences HCC cell motility, we examined the epithelial marker E-cadherin and the mesenchymal markers vimentin and ICAM-1. Western blot analysis demonstrated that when miR-615-5p was overexpressed, the protein level of E-cadherin was up-regulated by 1.39-fold, whereas vimentin and ICAM-1 were downregulated by 48% and 29%, respectively (Figure 4C). These data demonstrated that miR-615-5p represses the migration and invasion of HCC cells by regulating the EMT process.

Adhesion to a target tissue is considered to be one of the most critical steps in the invasive process for metastatic tumor cells. We investigated whether miR-615-5p can also affect HCC cell adhesion. According to our experimental data, adhesion activity was reduced by approximately 28%, 19% and 26% at 30 min, 60 min and 90 min, respectively, after overexpression of miR-615-5p in QGY-7703 cells (Figure 4D). Similar results were observed in HepG2 cells (Figure 4D). Next, we detected the protein level of β1-integrin in HCC cells, which is known to be associated with cell-matrix adhesion. Interestingly, the protein level of β1-integrin was decreased after transfected with pri-miR-615 (Figure 4E). These results indicate that miR-615-5p may affect HCC cell adhesion by regulating the β1-integrin signaling pathway.

To investigate whether miR-615-5p influences vasculogenesis, we performed a VM formation assay. As is shown in Figure 4F, the number of VM channels of the miR-615-5p overexpression group was fewer than that observed in the control group. Conversely, downregulation of miR-615-5p promoted the formation of VM channels (Figure 4F).

Collectively, miR-615-5p can suppress motility, adhesion and vasculogenic mimicry of HCC cells.

### miR-615-5p down-regulates RAB24

To identify the downstream molecules through which miR-615-5p affects the activities of HCC cells, we predicted the candidate target genes of miR-615-5p. Among the potential targets, we were interested in RAB24, which is involved in cytoskeletal remodeling, motility, and adhesion.

**Figure 5:**
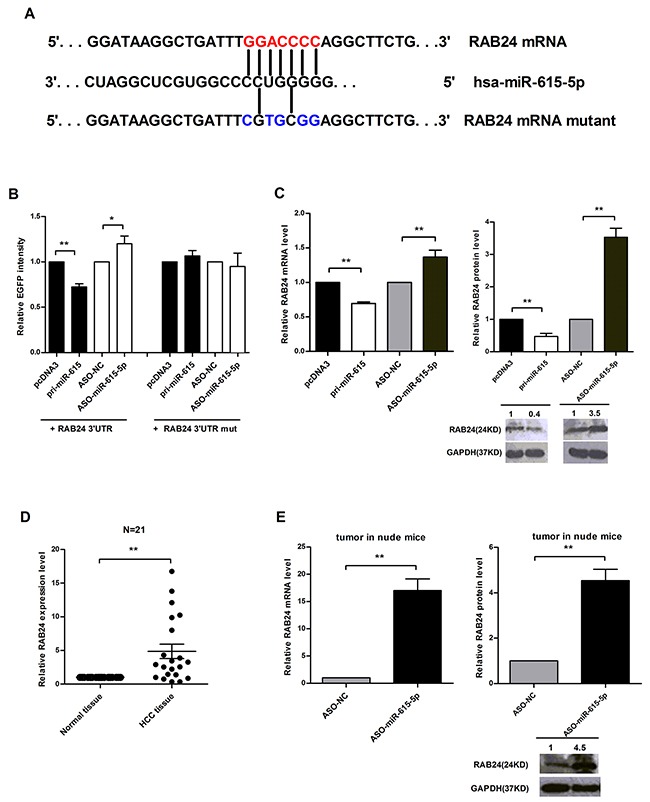
Identification of RAB24 as a direct target of miR-615-5p **A**. Predicted miR-615-5p binding sites in the 3’UTR of RAB24. **B**. The RFP plasmid was co-transfected as a normalization control. **C**. The mRNA and protein levels of RAB24 in QGY-7703 cells. **D**. The mRNA level of RAB24 in 21 pairs of HCC tissues was measured by qRT-PCR, β-Actin was used for normalization. Error bar indicate mean±SEM, **, p<0.01. **E**. qRT-PCR or western blot was used to detect the mRNA or protein levels of RAB24 in the metastatic nodules of the nude mice. β-Actin or GAPDH was used for normalization. Error bars in (B), (C) and (E) indicate the mean±SD of three independent experiments. *, p<0.05, **, p<0.01.

Base-pairing complementation revealed that the 3’ untranslated region (UTR) of RAB24 contains a putative binding region that is conserved among species and has significant complementarity with the seed sequence of miR-615-5p (Figure 5A). To confirm that miR-615-5p can bind to this region, a human RAB24 3’UTR fragment was cloned downstream of the EGFP reporter gene. The pcDNA3/EGFP-RAB24 3’UTR was then co-transfected with pri-miR-615 or ASO-miR-615-5p into QGY-7703 cells. It was found that pri-miR-615 reduced the fluorescence intensity by 28% compared with the control vector, but depletion of miR-615-5p increased the fluorescence intensity (Figure 5B). To further determine the necessity of this conserved binding site, we also constructed a vector with a RAB24 3’UTR that contained mutated miR-615-5p binding sites. Neither overexpression nor inhibition of miR-615-5p had a significant effect on the fluorescence intensity in cells transfected with the pcDNA3/EGFP-RAB24 3’UTR mutant (Figure 5B). Taken together, these data indicate that miR-615-5p binds directly to the 3’UTR of RAB24 to suppress its gene expression.

Next, we determined whether miR-615-5p regulates endogenous RAB24 expression. In QGY-7703 cells, ectopic expression of miR-615-5p led to a 31% or 53% decrease in the level of RAB24 mRNA or protein (Figure 5C). To verify the results obtained in cell lines, we also examined the expression level of RAB24 in 21 pairs of human HCC tissues. The result revealed that RAB24 was generally expressed at a higher level (Figure 5D), whereas miR-615-5p was expressed at a lower level (Figure 1A). To verify whether the negative correlation between miR-615-5p and RAB24 also exists in our mouse xenograft model, we isolated RNA and total protein of metastatic tumors in nude mice and found that the mRNA and protein level of RAB24 increased when miR-615-5p expression was blocked (Figure 5E), indicating that RAB24 is a direct and functional target of miR-615-5p.

### RAB24 promotes the malignant phenotype of HCC cells

**Figure 6:**
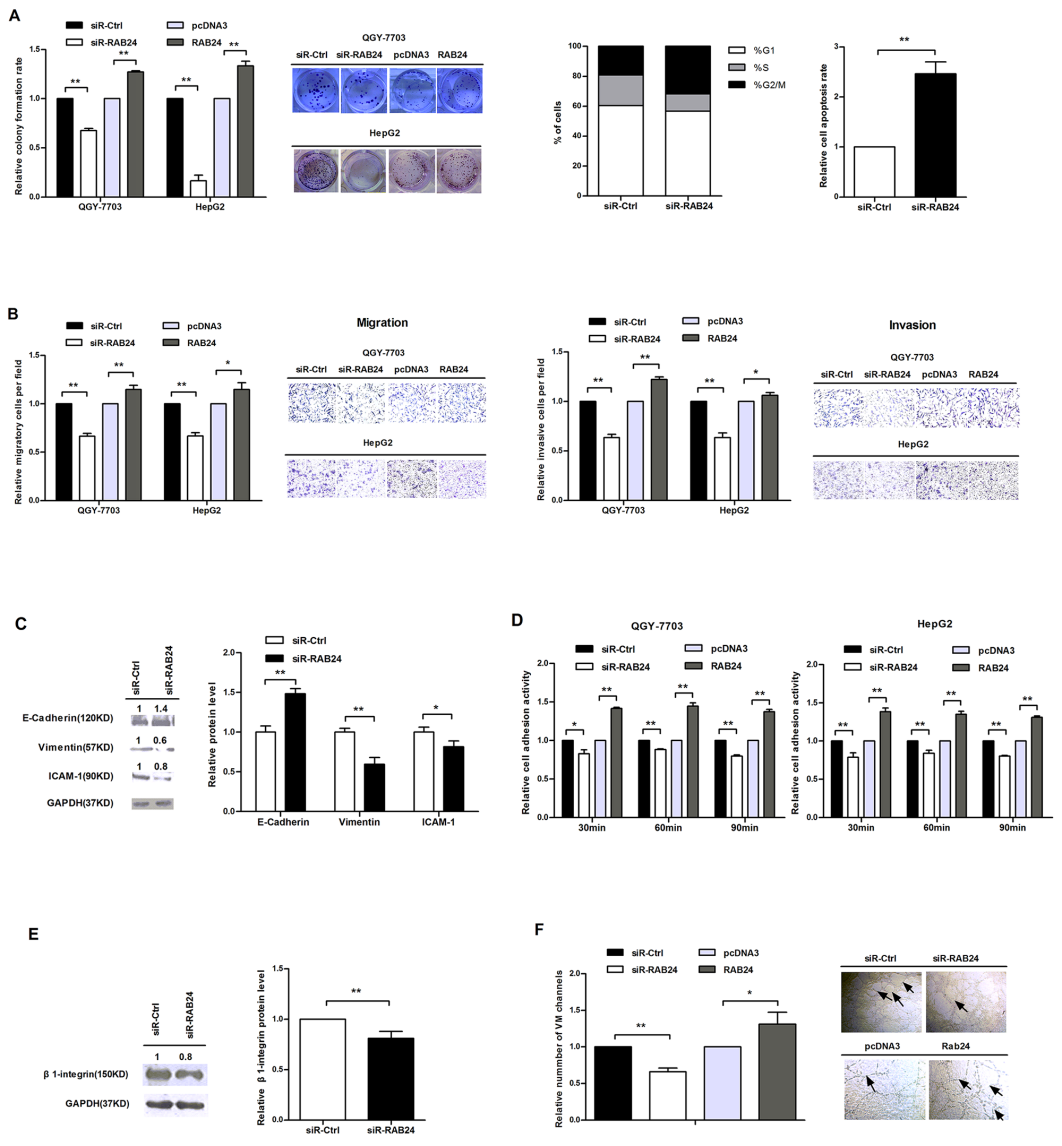
RAB24 promotes HCC cell growth, migration, invasion, adhesion and vasculogenic mimicry in vitro **A**. The effects of RAB24 knockdown or overexpression on cell growth (left), cell cycle (middle) and apoptosis (right). **B**. The influence of RAB24 on HCC cell migration (left) and invasion (right). **C**. The protein expression levels of E-cadherin, vimentin and ICAM-1 in QGY-7703 cells after knockdown of RAB24. **D**. Cell adhesion assay was performed as previously described. **E**. The protein level of β1-integrin in QGY-7703 cells when RAB24 expression was blocked. **F**. Vasculogenic mimicry assay was performed as previously described. Error bars in (A-F) indicate the mean±SD of three independent experiments. *, p<0.05, **, p<0.01.

Because RAB24 is a direct target of miR-615-5p, we wanted to determine the function of RAB24 in vitro. We constructed a vector (pSilencer/shRNA-RAB24, siR-RAB24) expressing a small interfering RNA (siRNA) targeted against RAB24 and another vector for RAB24 overexpression (pcDNA3-RAB24, RAB24). Before performing the functional studies, we first validated the efficiency of these vectors by western blot (Supplementary Figure S6). Next, we measured the effect of RAB24 on HCC cell growth, migration, invasion, adhesion and VM. Ectopic expression of RAB24 promoted HCC cell growth by accelerating cell cycle progression and reducing the rate of apoptosis (Figure 6A). As observed in the transwell migration and invasion assay (Figure 6B), overexpression of RAB24 facilitated the motility of HCC cells by regulating critical biomarkers of the EMT process (Figure 6C). Moreover, RAB24 promoted HCC cell adhesion by regulating the β1-integrin signaling pathway (Figure 6D and 6E), and promoted the VM formation of QGY-7703 cells (Figure 6F). On the contrary, knockdown of RAB24 suppressed the malignant activity of HCC cells. These results suggest that the role of RAB24 is opposite to that of miR-615-5p in HCC cells.

### Restoration of RAB24 counteracts the miR-615-5p-mediated inhibition of malignant phenotypes in HCC cells

**Figure 7:**
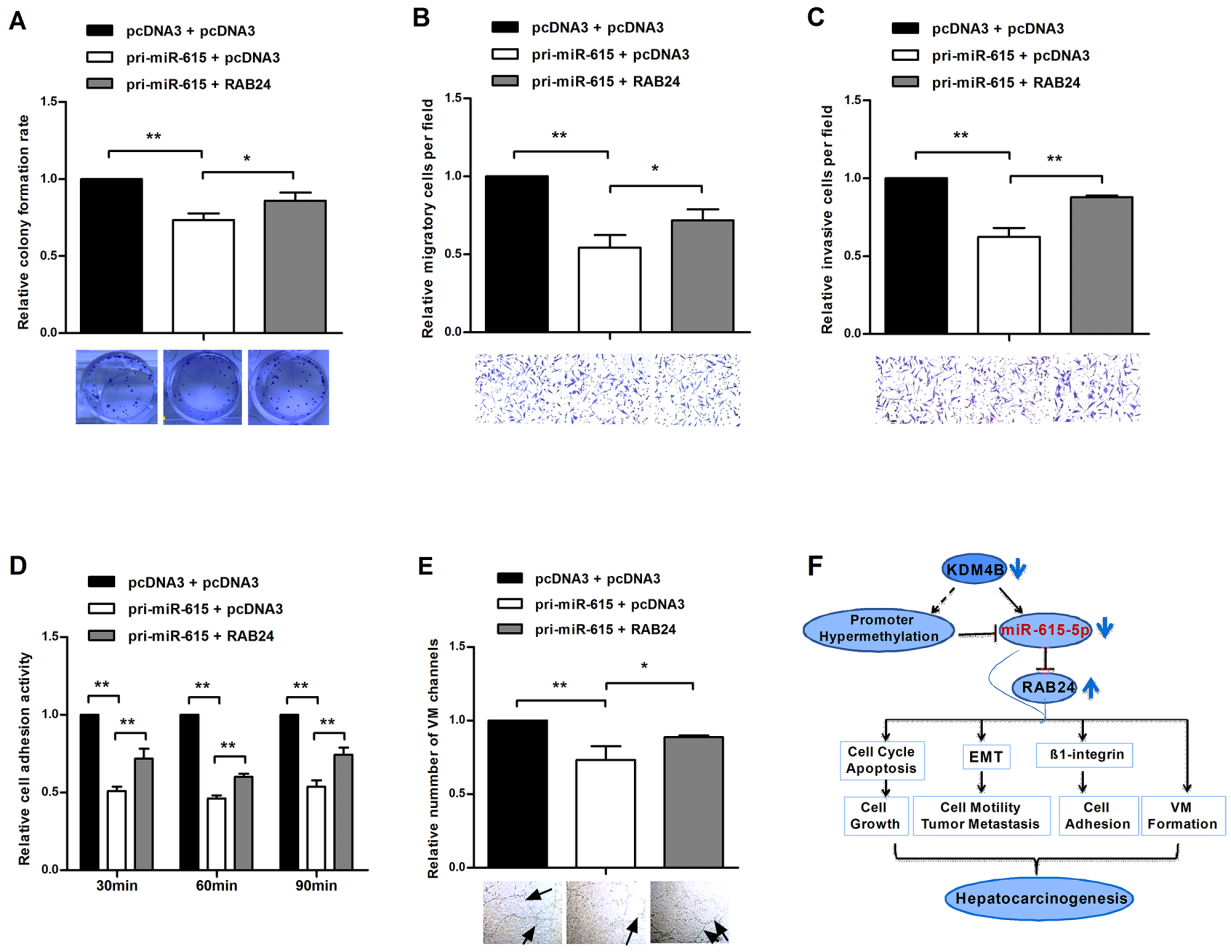
Restoration of RAB24 counteracts the miR-615-5p-mediated inhibition of HCC cell growth, migration, invasion, adhesion and vasculogenic mimicry **A-E**. Pri-miR-615 was transfected with or without RAB24. The colony formation assay (A), transwell migration (B) and invasion (C) assay, adhesion assay (D) and vasculogenic mimicry assay (E) were performed in the rescue experiment. **F**. Schematic representation of the role of miR-615-5p in the regulatory network of HCC. Error bars in (A-E) indicate the mean±SD of three independent experiments. *, p<0.05, **, p<0.01.

To confirm that the phenotype caused by miR-615-5p is mediated by the repression of RAB24, a rescue experiment was performed. QGY-7703 cells were transfected with pri-miR-615 plus RAB24 or empty pcDNA3. Ectopic expression of RAB24 promoted growth, migration, invasion, adhesion and vasculogenic mimicry in HCC cells, abrogating the inhibitory effect on malignant phenotype caused by miR-615-5p (Figure 7A-7E). These results indicate that miR-615-5p make its function, at least partially, by regulating RAB24 in HCC.

## DISCUSSION

Epigenetic aberrations regulate both protein-coding genes and non-coding RNAs, such as miRNAs [11]. These modifications may cause the inactivation of tumor-suppressive miRNAs and induce the initiation and progression of human cancer [12]. DNA methylation is the most important form of epigenetic regulation in eukaryotic cells [13]. To our knowledge, only a limited number of miRNAs have been illustrated to be silenced by DNA hypermethylation in hepatocellular carcinioma, including miR-1, miR-101, miR-124, miR-203 and miR-335 [14–17]. Here, we reported that miR-615-5p was downregulated by DNA hypermethylation in HCC tissues and cell lines. W Gao. et al demonstrated that miR-615-5p is epigenetically inactivated and functions as a tumor suppressor in pancreatic ductal adenocarcinoma [18]. However, miR-615-5p is epigenetically activated in prostate cancer cells [19], and it is not regulated by DNA methylation in the K-562 cell line [20]. One possible explanation is that miRNA expression depends on the cellular context [21], and the methylation status of genes is gene- and tumor-type-specific [22]. For example, miR-10a is up-regulated in hepatocellular carcinoma [23] and down-regulated in gastric cancer [24].

It is well known that DNA methylation and histone lysine methylation are closely associated with each other and affect the establishment of gene silencing patterns [25]. KDM4B, also known as JMJD2B, has been reported to demethylate H3K9me3 at pericentric heterochromatin in mammalian cells[26]. However, the role of KDM4B in euchromatin has not yet been illustrated. According to our results of genomic bisulfite sequencing, KDM4B, rather than DNMT1, DNMT3a, KDM4A, KDM4C, KDM5A or KDM6A, had a significant effect on the demethylation of the miR-615-5p promoter in HCC cells. KDM4B may mediates miR-615-5p CpG demethylation either directly through its interaction with the promoter DNA or indirectly through demethylation of H3K9.

Maniotis et al [27] first described a new type of vascularization, VM, in 1999. During VM, tumor cells form extracellular matrix (ECM)-rich, vasculogenic-like networks to complement the endothelial-cell-dependent vasculature, providing a space for the inflow of blood during tumor growth and metastasis. To date, researchers have discovered many proteins that could influence VM in aggressive HCC, including Slug, Osteopontin, HIF-1α, MMP-2 and Twist 1 [28–31]. However, little is known about the significance of miRNAs in the process of VM in HCC cells. We found that miR-615-5p could obviously reduce the number of VM channels of QGY-7703 cells in vitro. However, the detailed mechanism requires further investigation.

To date, only four miRNAs (miR-194, miR-124, miR-200c and miR-10a) have been demonstrated to regulate the EMT in liver cancer [23, 32–34]. Intriguingly, overexpression of miR-615-5p increased the protein level of the epithelial marker and decreased the levels of the mesenchymal markers, demonstrating the inhibitory effect of miR-615-5p on EMT. Many transcription factors, such as Snail, Slug, Zeb and Twist, participate in regulating the expression of EMT-associated markers [35]. Meanwhile, some of these transcription factors also influence the process of VM [28–31]. Therefore, the EMT and VM may be two mutually stimulatory biological phenomena during tumorgenesis.

We identified RAB24 as a direct target of miR-615-5p. Contrary to miR-615-5p, RAB24 is upregulated in HCC and promotes the malignant phenotype and EMT process of HCC cells. It has been reported that RAB24 was involved in the degradation of misfolded cellular proteins [36] and the genesis of the autophagosome[37, 38]. However, our present work provides the first evidence of the role of RAB24 in EMT and hepatocarcinogenesis. The impact of miR-615-5p and RAB24 on cell cycle is different. One possible explanation is that one miRNA may regulates many target genes, and one gene may targeted by many miRNAs. The integrins are a major familiy of cell adhesion receptors, and the integrin β1 subunit is crucial for adhesion to fibronectin [39]. Here, we demonstrated that miR-615-5p also suppressed the adhesion of HCC cells. Ectopic expression of miR-615-5p or knockdown of RAB24 suppressed HCC cell adhesion by reducing the protein level of β1-integrin.

Taken together, our data revealed that miR-615-5p functions as a tumor suppressor that is epigenetically silenced by downregulation of KDM4B in HCC. The silencing of miR-615-5p relieves the suppression of RAB24 and results in tumor growth, metastasis and VM (Figure 7F). These findings expanded our understanding of the regulatory network of miR-615-5p and the molecular mechanism that underlie hepatocarcinogenesis and metastasis, potentially providing new biomarkers for HCC.

## MATERIALS AND METHODS

### miRNA microarray analysis

A total of 640 DNA oligonucleotide probes from the mirVana miRNA Probe Set (Ambion) were designed according to the sequence of their mature miRNA. For more detail information, please see the Supplementary Materials and Methods.

### Clinical HCC specimens and cell lines

Thirty pairs of HCC samples and their corresponding adjacent normal hepatic tissues were obtained from the Cancer Center of Sun Yat-sen University of Medical Sciences. All of the samples were obtained with the patients’ informed consent, and the protocols were approved by the ethics committee. The clinical information of the patients was shown in Supplementary Table S5. QGY-7703 or HepG2 cells were cultured in RPMI-1640 (GIBCO) supplemented with 10% fetal bovine serum or MEM-α supplemented with 20% fetal bovine serum and 100 IU/ml penicillin and 100 mg/ml streptomycin. The cells were incubated at 37°C in a humidified chamber with 5% CO2. Cell transfection was performed with Lipofectamine Reagent (Invitrogen, Carlsbad, CA, USA) according to the manufacturer's protocol. These two human HCC cell lines express low levels of miR-615-5p, and they were stably cultured, passaged, and transfected in our laboratory. Therefore, we selected these two cell lines for the functional studies. L-O2 cells are a normal human liver cell line and were cultured using the same method employed for QGY-7703 cells.

### qRT-PCR and western blot

The large RNA and small RNA fractions from the tissue samples and cell lines were isolated using the mirVana™ miRNA Isolation Kit (Ambion, Austin, TX), according to the manufacturer's instructions. qRT-PCR was performed to detect the mRNA level of miR-615-5p, RAB24 and KDM4B. Western blot was performed to detect the protein level of RAB24, β1-integrin and EMT markers. The detailed procedures were described in Supplementary Materials and Methods and the primers used in qRT-PCR were shown in Supplementary Table S3.

### Prediction of the miR-615-5p promoter

Using the Promoter 2.0 Prediction Server (http://www.cbs.dtu.dk/services/Promoter/) and Promoter Scan (http://www-bimas.cit.nih.gov/molbio/proscan/), we identified the overlapping region as the putative promoter of miR-615-5p.We inserted this region upstream of the reporter gene in the pGL3-basic/luciferase vector and tested the luciferase activity following the instructions provided by the Dual-Luciferase Reporter Assay system (Promega, USA). We also used MethPrimer (http://www.urogene.org/methprimer/) to predict the CpG islands in the promoter region.

### Genomic bisulfite sequencing

For the demethylation experiments, cells were treated with 5 μmol/L 5-aza-2-deoxy-cytidine (5-Aza-CdR, Sigma) for 72 h, and the drug and medium were replaced every 24 h. Cells treated with DMSO were used as a control. Genomic DNA was extracted using the All Prep DNA/RNA Kit (Qiagen). One microgram of total genomic DNA was treated with sodium bisulfite according to the manufacturer's instructions provided with the EpiTect Kit (Qiagen). The CpG islands were amplified from the bisulfite-converted DNA by PCR, and the PCR products were cloned and sequenced.

### Construction of expression vectors

See the Supplementary Materials and Methods section for more detail. The oligonucleotides used in vector constructions were shown in Supplementary Table S4.

### Cells experiment

For detail information, please see Supplementary Materials and Methods.

### In vivo metastasis assay

In total, 1×10^6^ QGY-7703 cells were transfected with ASO-miR-615-5p or ASO-NC and suspended in 40 μl of serum free RPMI 1640/Matrigel (1:1) for each nude mouse. The cells were injected directly into the upper pole of the spleen of 5 to 6 week old female BALB/c-nu/nu mice. The mice were sacrificed 6 weeks later, and their spleens and livers were harvested. All procedures were performed according to the American Association for the Accreditation of Laboratory Animal Care guidelines for the humane treatment of animals and adhered to national and international standards.

### Statistical analysis

The differences in the results obtained in the colony formation assay, migration/invasion assay, cell adhesion assay and the luciferase assay were analyzed by two-tailed Student's t-test. The data are expressed as the means ± standard deviation (SD) of at least three independent experiments, and P≦0.05 was considered to be statistically significant. The correlations between miR-615-5p expression and methylation, KDM4B expression and methylation, and KDM4B expression and miR-615-5p expression, were analyzed by Spearman's rank correlation.

## SUPPLEMENTARY MATERIALS AND METHODS, FIGURES AND TABLES


