# Supplementary material for: KDM4B-mediated epigenetic silencing of miRNA-615-5p augments RAB24 to facilitate malignancy of hepatoma cells
Source: Oncotarget. 2016 Jul 25;8(11):17712–25. doi: 10.18632/oncotarget.10832 (PMC5392280; doi:10.18632/oncotarget.10832)
Supplement: Supplementary file 1 [file oncotarget-08-17712-s001.pdf]

KDM4B-mediated epigenetic silencing of miRNA-615-5p augments RAB24 to facilitate malignancy of hepatoma cells

SUPPLEMENTARY MATERIALS AND METHODS

miRNA microarray analysis

The probes were resuspended in 3× saline sodium citrate (SSC) at a concentration of 50 mM and spotted onto MICROMAX Glass Slides SuperChipI (PerkinElmer, MA, USA) at 50~60% humidity with the SpotArray 24 Microarray Printing System (PerkinElmer). Each probe was spotted in triplicate. Small RNAs were labeled with Cy5 or Cy3 dye (Amersham Biosciences, Piscataway, NJ, USA) using the mirVana miRNA Labeling Kit (Ambion). After hybridization at 42°C overnight (12-16 h), the slides were washed with SSC and scanned with a ScanArray Express Microarray Scanner using ScanArray 3.0 software (PerkinElmer).

qRT-PCR and western blot

To detect the expression level of miR-615-5p, small RNA (2 μg) was reverse transcribed into cDNA using M-MLV reverse transcriptase (Promega, Madison, WI, USA) with the primers listed in Supplementary Table 3. Both the mature miR-615-5p and the endogenous control U6 snRNA were amplified by PCR using this cDNA. Specific forward primers and a universal reverse primer were used as shown in Supplementary Table 3. The PCRs were performed under following conditions: 94°C for 4 min, followed by 40 cycles of 94°C for 30 s, 56°C for 30 s, and 72°C for 30 s. These reactions were performed with SYBR Premix Ex Taq (TaKaRa, Otsu, Shiga, Japan) using the iQ5 Real-Time PCR Detection System (Bio-Rad).

To detect the relative levels of RAB24 transcripts, large RNA (5 μg) was reverse transcribed into cDNA using M-MLV reverse transcriptase (Promega, Madison, WI, USA). The RAB24 gene and the β-actin gene, which served as an endogenous control, were amplified from the cDNA using the primers shown in Supplementary Table 3. Real-time PCR was performed as described above. The detection of KDM4B is the same with RAB24. The primers are shown in Supplementary Table 3.

In western blot analysis, cultured cells were washed with PBS and lysed at 4°C for 30 min by RIPA Buffer. All collected proteins were resolved on a SDS-denaturing polyacrylamide gel and then transferred onto a nitrocellulose membrane. Membranes were incubated with blocking buffer for 2 hours at room temperature and then incubated overnight at 4°C with an anti-RAB24 (anti-E-Cadherin, anti-Vimentin, anti-ICAM-1, anti-β1-integrin) antibody or anti-glyceraldehyde phosphate dehydrogenase (GAPDH) antibody (Tianjin Saier Biotech, Tianjin, China) mixed in blocking buffer. Then the membranes were washed and incubated with a horseradish peroxidase-conjugated secondary antibody. Protein expression was assessed by enhanced chemiluminescence and exposure to chemiluminescent film. The band intensities were quantified by LabWorks image acquisition and analysis software (UVP, Upland, CA, USA). The protein level of the control group was defined as 1.

Construction of expression vectors

To construct the pcDNA3/pri-miR-615 (pri-miR-615) expression vector, we amplified a 266-bp DNA fragment carrying the miR-615-5p precursor from genomic DNA by PCR and inserted it into the pcDNA3 vector between the KpnI and EcoRI sites. We also commercially synthesized a 2'-O-methyl-modified antisense oligonucleotide (ASO-miR-615-5p) to inhibit the function of miR-615-5p.

To construct the enhanced green fluorescent protein (EGFP) reporter plasmid, the coding sequence of EGFP was subcloned into the pcDNA3.1 multiple cloning site to form the pcDNA3-EGFP vector. The wild-type and mutant forms of the RAB24 mRNA 3'UTR were amplified by PCR from a QGY-7703 cDNA library and subsequently inserted downstream of the EGFP stop codon between the BamHI and EcoRI sites.

The pSilencer/sh-RAB24 (siR-RAB24) vector was constructed by annealing the sense and antisense strands of encoding a hairpin RNA and inserting the fragment into the pSilencer2.1 neo vector (Ambion) between the BamHI and HindIII sites. To construct the RAB24 ectopic expression vector(without its 3'UTR), the coding sequence of RAB24 was amplified by PCR from a cDNA library derived from QGY-7703 cells and cloned into the pcDNA3 vector at the EcoRI and XhoI sites to form the RAB24 expression vector pcDNA3/RAB24(RAB24). The DNMT1, DNMT3a, KDM4A, KDM4B, KDM4C, KDM5A and KDM6A overexpression vectors were purchased from Addgene (Cambridge, MA, USA).

All insertions described above were verified by DNA sequencing. All the primers are shown in Supplementary Table 4.

Colony formation assay and cell adhesion assay

For the colony formation assay, QGY-7703 or HepG2 cells were counted and seeded in 12-well plates (in triplicate) after transfection at 200 or 800 cells per well,

respectively. The culture medium was replaced every 3 days. Colonies were counted only if they contained more than 50 cells, and the number of colonies was counted either 7 days (QGY-7703 cells) or 15 days (HepG2 cells) after seeding. The colony formation rate was calculated using the following equation: colony formation rate = (number of colonies/number of seeded cells) × 100%.

For the cell adhesion assay, QGY-7703 (5×10^5/well) or HepG2 (8×10^5/well) cells were added into 96-well plates coated with Matrigel (0.2 mg/ml). At 30, 60, and 90 min after seeding the floating cells were removed by rinsing the wells with PBS. Then, MTT was added to each well, and the plates were incubated for 4 h. The absorbance at a wavelength of 570 nm was used to calculate the number of the adherent cells.

Cell cycle and apoptosis analysis

Transfected QGY-7703 cells were seeded in 6-well plates in duplicate for 24 h in complete culture solution. One group of cells was deprived of serum for 24 h before harvesting, while another group of cells was returned to complete medium for another 24 h before harvesting. Cells were gathered by centrifugation, fixed in 95% (V/V) ethanol. The DNA content of QGY-7703 cells was analyzed with FACS Calibur flow cytometer (BD Biosciences) and Cell Quest software (BD Biosciences). Before the analysis wash the cells with phosphate-buffered saline (PBS), resuspend them in propidium iodide (PI) staining buffer (PBS, 0.1% Triton X-100, 60 μg/ml PI, 0.1 mg/ml DNase-free RNase, and 0.1% trisodium citrate) for 30 minutes on ice.

The terminal deoxynucleotidyl transferase-mediated dUTP nick end-labeling (TUNEL) assay was performed according to the instruction of the In-Situ Cell Death Detection Kit (Roche Diagnostics Corporation, Indianapolis, IN, USA). 5000 transfected QGY-7703 cells were planted into 14-well plates in triplicate. Then, treat the cells with 0.5 ppc paclitaxel for 1 h, fix them in paraformaldehyde (4% in PBS, pH 7.4), incubated the plates in permeabilization solution (0.1% TritonX-100 in 0.1% sodium citrate) for 2 minutes on ice, add 4μl of TUNEL reaction mixture (0.4μl of the enzyme solution and 3.6μl of the labeling solution) to each well, incubate the slide at 37°C for 1 h in the dark. After that, TUNEL reactions were performed follow the manufacturer's protocol. Fluorescence images were observed with a Nikon Digital sight DS-U1 scanning microscope (Nikon, Tokyo, Japan).

Transwell migration and invasion assays

For the transwell migration assay, 6×10^4 QGY-7703 cells or 12×10^4 HepG2 cells were placed in the upper chamber of each insert (Corning, Cambridge, USA) containing the non-coated membrane. For the invasion assay, 6×10^4 QGY-7703 cells or 12×10^4 HepG2 cells were placed on the upper chamber of each insert coated with 40 μl of Matrigel (Clontech, Mountain View, CA) diluted to 4 μg/μl with RPMI 1640 medium for the QGY-7703 cells or to 1 μg/μl with MEM-α medium for the HepG2 cells. An 800 μl volume of medium supplemented with 20% fetal bovine serum was added to the lower chambers. For the migration assay, the QGY-7703 cells were evaluated after 12 h, and the HepG2 cells were evaluated after 48 h. For the invasion assays, the QGY-7703 cells were evaluated after 12 h, and the HepG2 cells were evaluated after 72 h. For the evaluation of both the migration and invasion assay, cells attached to the lower surface were stained for 20 min with crystal violet and then photographed for counting.

Vasculogenic mimicry formation assay

The vasculogenic mimicry (VM) experiments were performed just as follows. Matrigel (10 mg/ml, Clontech, Mountain View, CA) was placed at 4°C overnight to form a gel. Then, 40 μl of Matrigel was dispensed onto 24-well tissue culture plates and subsequently incubated at 37°C for 2 h for solidification. Then, 1.5×10^5 QGY-7703 cells were seeded onto the coated well 24 h after transfection. After growth for 24 h at 37°C, VM was assessed using an inverted microscope (OLYMPUS, Japan).

Fluorescent reporting assay for miRNA target identification

To confirm the direct interaction between miR-615-5p and RAB24 mRNA, EGFP reporter plasmid and pcDNA3-pri-miR-615, pcDNA3, ASO-miR-615-5p or ASO-NC were transiently cotransfected into QGY-7703 cells. The RFP expression vector pDsRed2-N1 (Clontech, Mountain View, CA) was used for normalization. The EGFP and RFP fluorescence levels were measured with an F-4500 Fluorescence Spectrophotometer (Hitachi, Tokyo, Japan).

SUPPLEMENTARY FIGURES AND TABLES

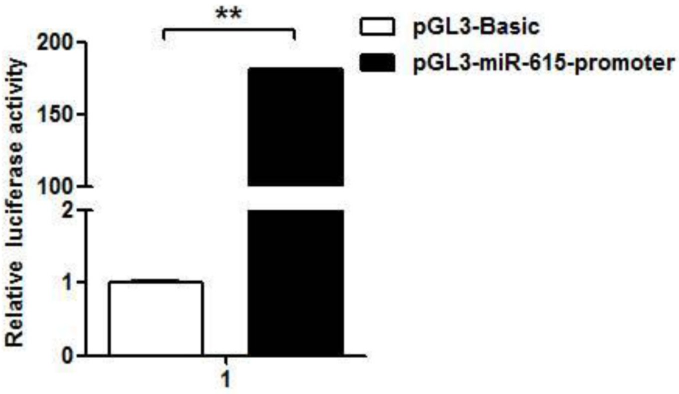

Supplementary Figure S1: Construction of the miR-615-promoter vector. The luciferase reporter assay showed that the fragment that we inserted upstream of the reporter gene had strong promoter activity.

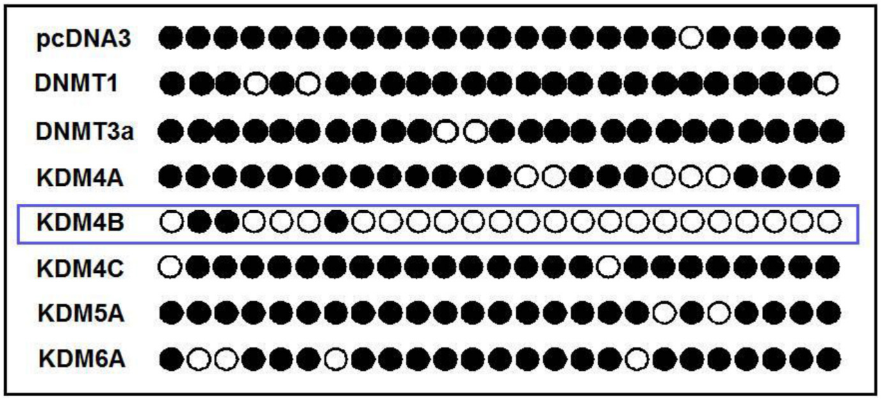

Supplementary Figure S2: Screening for the potential enzyme responsible for the hypermethylation of the miR-615 promoter. Vectors for the overexpression of DNMT1, DNMT3a, KDM4A, KDM4B, KDM4C, KDM5A and KDM6A were transfected into QGY-7703 cell. However, only ectopic expression of KDM4B significantly reduced the methylation of CpG loci according to genomic bisulfate sequencing.

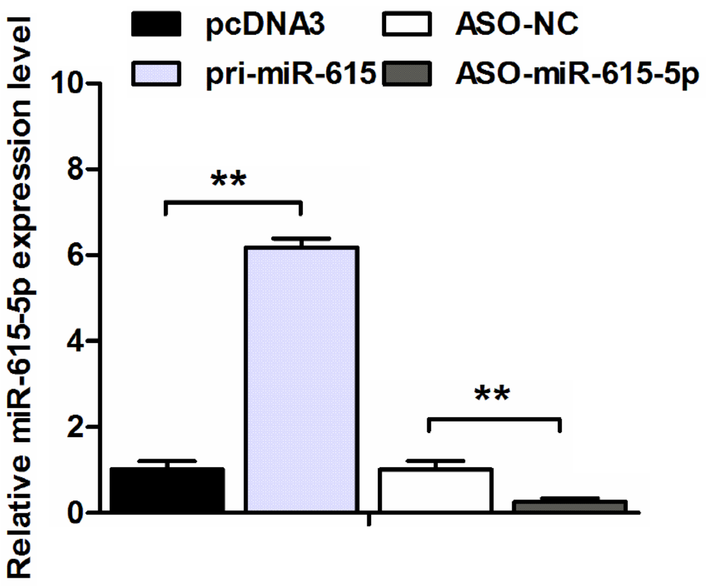

Supplementary Figure S3: Efficiency of the pri-miR-615 and ASO-miR-615-5p plasmids. qRT-PCR was performed to detect the mRNA level of miR-615-5p after transfecting pri-miR-615 or ASO-miR-615-5p into QGY-7703 cells. The two plasmids effectively increased or decreased the expression of miR-615-5p. *, p<0.05, **, p<0.01.

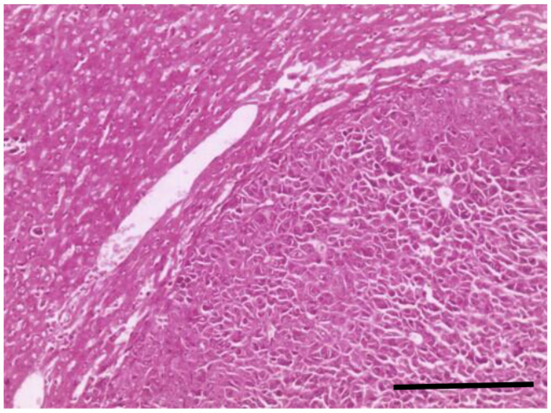

Supplementary Figure S4: H-E staining reveals the interface of the metastatic tumor and the adjacent liver tissue. Bar, 200 μm.

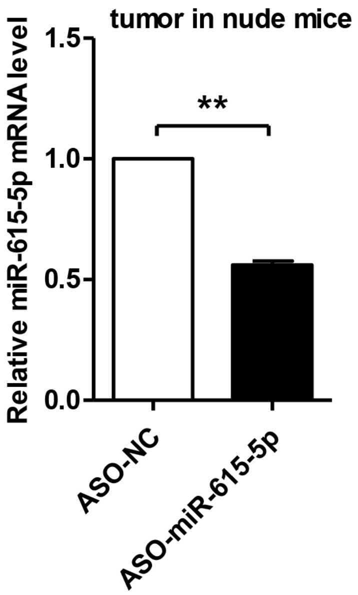

Supplementary Figure S5: qRT-PCR was performed to confirm the expression level of miR-615-5p in metastatic tumors of both groups.

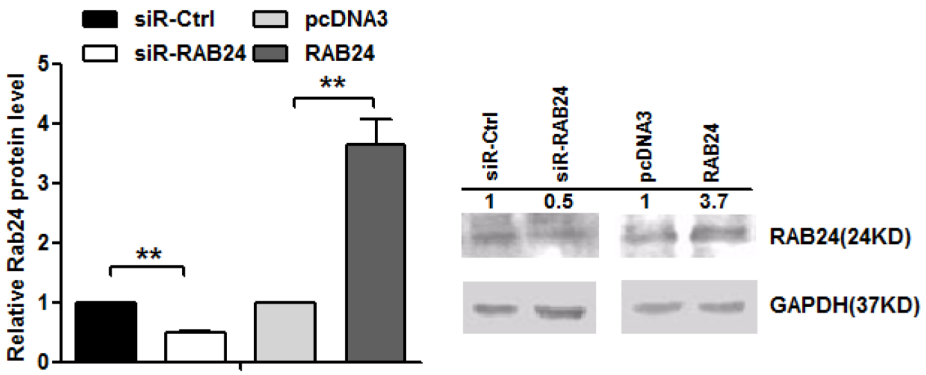

Supplementary Figure S6: Effectiveness of the siR-RAB24 and RAB24 vectors. Western blot analysis of RAB24 expression in QGY-7703 cells after knockdown or overexpression of RAB24. The two plasmids, siR-RAB24 and RAB24, effectively increased and decreased the RAB24 protein level, respectively. GAPDH was used as an internal control for normalization.

Supplementary Table S1: miRNAs upregulated in microarray after treatment with 5-Aza-CdR

miRNA	Fold change	miRNA	Fold change	miRNA	Fold change
miR-506	1.91	miR-1272	5.93	miR-27a	18.00
miR-532-3p	2.58	miR-1274a/b	3.09	miR-29b-2	2.01
miR-561	8.53	miR-1287	2.98	miR-34a	14.22
miR-574-5p	1.22	miR-1290	10.01	miR-92-1	1.91
miR-598	7.02	miR-1293	4.79	miR-92-2	3.02
**miR-615-5p**	**46.08**	miR-1299	5.64	miR-101-1	1.90
miR-625	1.22	miR-1306	9.87	miR-122a	2.01
miR-629	6.98	miR-1826	7.93	miR-134	1.99
miR-639	3.00	miR-1915	2.31	miR-145	1.78
miR-642	3.51	let-7a-2	3.14	miR-146	2.01
miR-646	6.92	miR-15a	2.09	miR-182	2.00
miR-765	4.38	miR-18	4.03	miR-184	14.25
miR-941-1/2/3	4.32	miR-19a	1.54	miR-194-1	2.03
miR-1226	1.79	miR-19b-1	2.00	miR-200a	3.07
miR-1250	6.84	miR-24-2	1.65	miR-296	12.30
miR-1261	6.21	miR-25	7.36	miR-222	25.55

Supplementary Table S2: miRNAs downregulated in microarray after treatment with 5-Aza-CdR

miRNA	Fold change	miRNA	Fold change	miRNA	Fold change
miR-452	0.77	miR-1308	0.91	miR-30eHA	0.79
miR-483	0.87	miR-1974	0.59	miR-34b	0.64
miR-484	0.96	miR-1975	0.93	miR-141	0.30
miR-489	0.89	miR-1977	0.61	miR-150	0.48
miR-638	0.69	miR-2110	0.66	miR-192	0.49
miR-720	0.57	let-7a-1	0.80	miR-200b	0.52
miR-744	0.70	let-7b	0.91	miR-200c	0.39
miR-1280	0.68	miR-26a-1	0.74	miR-221	0.70
miR-1281	0.71	miR-26a-2	0.63	miR-320	0.87

Supplementary Table S3: The primers used in qRT-PCR

Name	Sequence (5' →3')
miR-615-5p RT	GTCGTATCCAGTGCAGGGTCCGAGGTGCACTGGATACGACCATCCGA
miR-615-5p Forward	TGCACGGGGGTCCCCGGTGCTCG
U6 RT	GTCGTATCCAGTGCAGGGTCCGAGGTGCACTGGATACGACAAAATATGG
U6 Forward	TGCGGGTGCTCGCTTCGGCAGC
Reverse	CCAGTGCAGGGTCCGAGGT
RAB24-qPCR-S	CAGCTCTTTGAAACATCCAGCAAG
RAB24-qPCR-A	CCAGATCCACGCCCTTGTCC
β- Actin-S	CGTGACATTAAGGAGAAGCTG
β- Actin-A	CTAGAAGCATTTGCGGTGGAC
miR-615-BSP-S	TGTAAATTTGTGGGAATTATGG
miR-615-BSP-A	ACTCCTCTTTAATCTCCCCACTA
KDM4B-qPCR-S	GTCATCACCAAGAACCGCAACG
KDM4B-qPCR-A	CAGTCCCTACTCGTGATGCTC

Supplementary Table S4: The oligonucleotides used in vector constructions

Name	Sequence (5' →3')[α]
miR-615-5p-promoter-S	GGAATGGTACCGAGGGCGCGCCTTGGCC
miR-615-5p-promoter-AS	GGCGGAGATCTTGGAATCCTCCGGAGCCGCAAG
pri-miR-615-S	GAGGAGGTACCAGAGGGATCTGAAGGGTGAGG
pri-miR-615-A	GAGGAGAATTCGCAGGAAACTTTCCACCGC
ASO-NC	CAGUACUUUUGUGUAGUACAA
ASO- miR-615-5p	GAUCCGAGCACCGGGGACCCCC
RAB24-3'-UTR-S	CTGGGATCCGTAGTCAAATGTCTGAGCTAC
RAB24-3'-UTR-A	CGGAATTCGGGTGTGATGCACAGTGC
RAB24-3'-UTR-MS	GGCTGATTTCGTGCGGAGGCTTCTG
RAB24-3'-UTR-MA	CAGAAGCCTCCGCACGAAATCAGCC
RAB24- siR-Top	GATCCGAGTGACCTGCTGGAAGAATTCAAGAGATTCTTCCAGCAGGTCACTCTT TTT TGGAAGAATTCA
RAB24- siR-Bot	AGCTTGAATTCTTCCAAAAAAGAGTGACCTGCTGGAAGAATCTCTTGAATTCTTC CAGCAGGTCACTCG
RAB24-S	CTGAATTCTCCACCATGAGCGGGCAGCGCGTG
RAB24-A	GAGTCCTCGAGCAGTGATGACAACAGCTGTAG

[α]Restriction sites are underlined

Supplementary Table S5: Clinicopathological data of HCC patients

Index	Gender	Age	Clinical diagnosis	TNM Stage
1	Male	20	HCC	T1N0M0
2	Female	68	HCC	T1N0M0
3	Male	45	HCC	T1N0M0
4	Male	30	HCC	T1N0M0
5	Male	46	HCC	T1N0M0
6	Male	55	HCC	T4N0M0
7	Male	36	HCC	T4N0M0
8	Male	38	HCC	T1N0M0
9	Male	35	HCC	T1N0M0
10	Male	54	HCC	T1N0M0
11	Male	44	HCC	T1N0M0
12	Male	42	HCC	T1N0M0
13	Male	34	HCC	T1N0M0
14	Male	34	HCC	T1N0M0
15	Male	34	HCC	T1N0M0
16	Male	35	HCC	T1N0M0
17	Female	65	HCC	T3N0M0
18	Female	51	HCC	T4N0M0
19	Male	40	HCC	T3N0M0
20	Male	36	HCC	T2N0M0
21	Male	50	HCC	T4N0M0
22	Male	53	HCC	T4N0M0
23	Male	41	HCC	T1N0M0
24	Female	55	HCC	T2N0M0
25	Male	69	HCC	T1N0M0
26	Female	38	HCC	T1N0M0
27	Male	36	HCC	T3bN0M0
28	Male	60	HCC	T2N0M0
29	Male	59	HCC	T2N0M0
30	Male	49	HCC	T1N0M0
